# The role of an educational vignette to teach dental students on issues of substance use and mental health disorders in patients at the University of British Columbia: an exploratory qualitative study

**DOI:** 10.1186/s12909-021-02767-9

**Published:** 2021-06-29

**Authors:** Mario Brondani, Rana Alan, Leeann Donnelly

**Affiliations:** 1 Department of Oral Health Science, Faculty of Dentistry, University of British Columbia, 2199 Wesbrook Mall, Vancouver, BC V6T 1Z3 Canada; 2 Smile Dental Center, Boston, MA USA; 3 Department of Oral & Biomedical Sciences, Faculty of Dentistry, University of British Columbia, Vancouver, Canada

**Keywords:** Substance use, Mental health disorders, Undergraduate dental education, Undergraduate dental hygiene education, Inverted classroom approach, Vignette, Reflections

## Abstract

**Background:**

Inverted classroom approaches and the use of vignettes have been suggested in health care education. The objective of this study was to use an educational vignette to discuss issues of stigma around substance use and mental disorders within undergraduate Doctor of Medicine in Dentistry (DMD) and Bachelor of Dental Science in Dental Hygiene (BDSc-DH) students at the University of British Columbia, Canada. Our research question was *“how can an educational vignette, depicting a fictitious patient with a history of substance use and mental health disorders accessing dental care, promote an open dialogue about stigma?”*

**Methods:**

An educational vignette was developed based on individuals’ lived-experiences with a variety of substance use and/or mental health disorders. This vignette was used to generate in-class discussion involving all the DMD and BDSc-DH undergraduate students enrolled between 2015/16 and 2018/19 who attended a mandatory 2.5 h didactic session using an inverted classroom approach. Students were also encouraged to provide a post-class voluntary written reflection, between 200 and 300 words, around stigma. The authors took written field notes on students’ response to the vignette and used excerpts from students’ de-identified reflections to illustrate the impact of such an educational tool.

**Results:**

A total of 323 DMD and BDSc-DH students attended the didactic sessions between 2015/16 and 2018/19, and 148 reflections were submitted over the same time period. The inverted classroom approached showed to be engaging and collaborative. The vignette promoted open dialogue and was determined to be a conducive tool to generate in-class discussion and reflection. Major themes from the textual data included ‘*exploring power relations’* and ‘*patient-centered care approach to counteract stigma’*. The vignette also enabled the discussion of positive experiences characterized by empathy, reassurance and communication, although it might not have prompted all students to participate in class or in writing the reflections.

**Conclusion:**

The inverted classroom approach and the vignette seemed to be an effective way to facilitate dialogue and reflection for most students. This study highlighted the need to explore innovative ways in which to continuously prepare current and future oral health care providers to professionally address the needs of patients with a history of substance use and/or mental health disorders.

## Background

Access to oral health care hinges upon the ability to afford the services [1, 2]. For those individuals with history of substance use and/or who suffer from mental health disorders, access to this care is further impacted by stigma they likely face by the providers and their staff [3, 4]. Stigma denotes a negative characteristic, from *a mark of disgrace* to experiences of *discrimination, prejudice* and *stereotyping* [5, 6]. Link and Phelan [5] further proposed a framework where stigma would exist when five intertwined components occur: labelling based on human differences, stereotyping associated with negative characteristics, social disqualifying or excluding, and diminishing the social status of these individuals by power imbalance [5]; such imbalance can be present in health care settings [7] and other aspects of one’s life [8, 9].

Reports point to a “*connection between mental illness and the use of addictive substances*” as mental health disorders seem to increase the use of addictive substances [10], although both conditions may not co-occur. Nonetheless, individuals with substance use and/or mental health disorders are more likely to be stigmatized and discriminated against by the public at large [11] and by health providers in particular compared to their counterparts [3, 12, 13]. Such stigma is manifested in a wide range of behaviours, from refusal of care [14], to unethical treatments and expressions of discomfort around patients [15] as experienced by those with HIV/AIDS [16], who are homeless [17], or who are of certain ethnicities [18].

Oral diseases are prevalent among those with substance use and/or with history of mental health disorders [19, 20], and can only worsen when needed care is postponed or forgone altogether due to health providers’ potential stigmatizing attitudes. One suggested approach to start addressing stigma and discrimination consists of discussing such topics with undergraduate^1^ Doctor of Medicine in Dentistry (DMD) and Bachelor of Dental Science in Dental Hygiene (BDSc-DH) before they became licenced health care professionals, as currently done at the University of British Columbia (UBC), in Canada. In turn, focus has been placed on graduating socially responsible oral health care providers who must balance the economic growth of their practices and the welfare of the community at large [21–23], and of those who are vulnerable and from special-needs populations in particular [24]. Some of the most successful educational approaches to promote stereotype change include presenting stories about individuals with the conditions of interest, directly exposing students to rightly stigmatized groups of patients, engaging the impacted community members as the teachers in the classroom, and incorporating self-reflection techniques in their educational training [23, 25, 26].

**Fig. 1:**
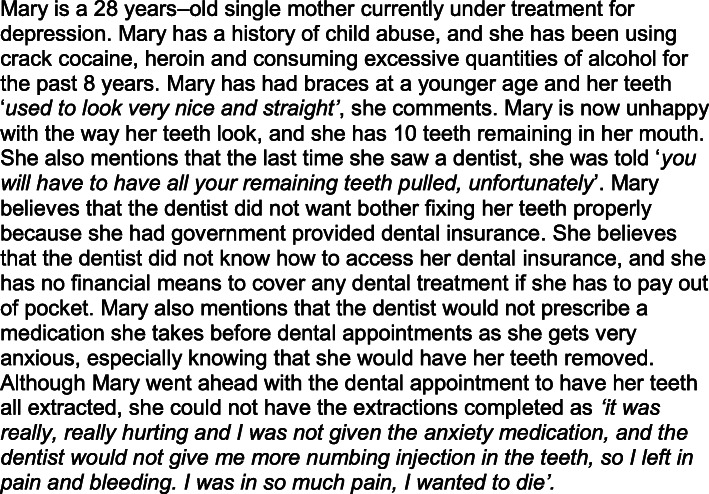
The educational vignette developed from interviews with patients with a history of substance use and mental health disorders

For this reason, previous qualitative research using interviews explored stigmatizing behaviours from dental professionals as experienced by individuals with a history of substance use and/or with mental health disorders [3, 12]. An educational vignette was developed from those interviews and is presented in Fig. 1. Vignettes encourage thoughtful explanations and potential solutions for the situation presented [27, 28], and are utilized when certain issues are difficult or sensitive for people to openly discuss, including oral disability within older adults [29], and mother-to-child transmission of HIV [30]. The vignette from Fig. 1 was developed as such so that gender, age, self-perceived aesthetics, dental insurance benefits, pain management and patient/provider relationship could be discussed within the context of substance use and/or mental health disorders. This vignette has been employed with undergraduate DMD and BDSc-DH students at the UBC [3, 12] within an inverted classroom approach^2^ [23, 31]. In turn, our research question was “how can an educational vignette, depicting a patient with a history of substance use and mental health disorders accessing dental care, promote an open dialogue about stigma?” More specifically, we aimed at determining how a context-based vignette could facilitate dialogue and be a conducive educational tool for discussing stigmatizing dental experiences of individuals with a history of substance use and/or mental health disorders. Our two outcomes were to explore the use of the vignette in promoting an open dialogue around stigma and around mental health disorders. The study reported herein was developed pre-COVID19 pandemic, where physical distancing within the classroom did not apply.

## Methods

After initially testing the clarity and comprehension of the vignette with a group of seven graduate students (MSc and PhD) in 2014 [12], we used the vignette from 2015/16 to 2018/19 with the first year of the undergraduate DMD and third year of the undergraduate BDSc-DH students at the UBC over a 2.5-h didactic mandatory session [25]. This session aims to address substance use and mental health disorders, and it is delivered annually with the two cohorts of students together in Term 2. The class size of undergraduate dental students varies from 47 to 60 students, and the undergraduate dental hygiene varies from 23 to 26 students, depending on the academic year [19, 25, 32].

This 2.5-h didactic session employs an inverted classroom approach where students had a required pre-reading pertaining to the session’s content to be discussed in class, a brief introduction about the pathophysiology of addiction in general, a testimony from community members experiencing substance use and/or mental health disorders, small group discussion about the vignette followed by a wrap up conversation, and a post-session volunteer reflection (Table 1).

**Table 1:** The flipped classroom approach to address substance use and mental health disorders in undergraduate dental and dental hygiene education

	Substance use and mental health disorders
**Pre-session**	Pre-reading: *‘Integrating issues of substance abuse and addiction within undergraduate dental education’* [30]
**2.5 h session** **(2:30-5 PM)**^a^	Definition of the terms *Community driven presentation* (40 min) - Q&A about the presentation in light of the pre-reading (15 min) Personal testimonial of community members with a history of substance use and/or mental health disorders (40 min) Vignette about Mary (5 min) In-class small group^b^ discussion about the vignette (40 min): * - How do you see the issues of access to care and acceptance within this scenario? From whose perspective?* * - Do you agree/disagree with both dentists’ actions? Why?* * - What would you do if you were her dentist?* * - What stigma means to you? Is there stigma portrayed on this scenario? Why and why not?* Wrap up with community members, instructor and students
**Post-session**	Voluntary self reflection^c^

^a^Instructors take verbatim notes of the students’ contribution to the in-class discussions

^b^Given the larger size of the lecture hall, students remain in class. Groups are arranged so that dental and dental hygiene students are represented in each group. Instructors walk around during the small group discussing to take verbatim notes

^c^Students are encouraged to self-reflect individually, after the session. Written reflection are kept between 200 and 300 words as students answer ‘how did this session make you fell?’ De-identified written reflections were shared with the authors

Students submitted their reflections individually in written form between 200 and 300 words, as they were asked to consider ‘how did this session make you feel?’ The UBC undergraduate students have experience in reflecting, an exercise that is promoted throughout all the academic years as students consider the impact and/or relevance of an experience, a lesson learned, a challenge faced, or an activity they performed; this exercise enhances knowledge and facilitates personal and professional growth [26].

Between 2015/16 and 2018/19, we had taken de-identified verbatim notes of students’ contributions during the session in general and during the in-class small group discussion of the vignette in particular. During these 4 years, all 219 dental and 104 dental hygiene students have attended this session as part of their mandatory curriculum (323 students in total, Table 2). All students were included in this study as they attended the above described session.

**Table 2:** Class composition and student demographics according to the academic year, gender, and cohort

Academic year	Gender: Male/Female N (%)	Cohort:^a^ DMD / DHYG N (%)	
2015/16	*Male – 27 (37.5)*	*DMD – 23 (85.2)* *DHYG – 4 (14.8)*	27
	*Female – 45 (62.5)*	*DMD – 24 (53.4)* *DHYG – 21 (46.6)*	45
			**72 (100)**
2016/17	*Male – 31 (35.2)*	*DMD – 28 (90.3)* *DHYG – 3 (09.7)*	31
	*Female – 57 (64.8)*	*DMD – 32 (56.1)* *DHYG – 25 (43.9)*	57
			**88 (100)**
2017/18	*Male – 32 (38.1)*	*DMD – 29 (90.9)* *DHYG – 3 (09.1)*	32
	*Female – 52 (61.9)*	*DMD – 27 (51.9)* *DHYG – 25 (48.1)*	52
			**84 (100)**
2018/19	*Male – 46 (58.2)*	*DMD – 30 (91.0)* *DHYG – 3 (09.0)*	**33**
	*Female – 33 (41.8)*	*DMD – 26 (56.5)* *DHYG – 20 (43.4)*	**46**
			**79 (100)**

^a^*DMD* Doctor of Medicine and Dentistry, *DHYG* Dental Hygiene

We present these data anonymized and only providing the student gender and year, and whether or not the student was from the undergraduate dental or dental hygiene cohort when excerpts from these notes are used. Accuracy of the answers and responses were kept verbatim as much as possible; coding analysis was not warranted in this study given its broader goal to readily portray the overall reactivity of the students about the session and the vignette – it is not a traditional qualitative study either. Rather, the de-identified data from the written notes and reflections were read by two authors (MB and LD) separately. An initial round of face-to-face interactive dialogue took place with the authors exploring five reflections and the notes taken in 2015/16 to confirm the main general themes and discus any discrepancy or conflict about the themes; this was also used as a calibration exercise. After, the remaining textual data were analyzed independently by the authors as described elsewhere [23, 26].

As we have employed in previous studies [13, 25, 29], the results are presented and readily discussed within the same section to optimise flow and connectivity of the information. We opted to combine results and discussion into one section to provide a cohesive presentation of the findings along with meaningful linkages to the existing literature, as suggested by Anderson [33] and Burrard and colleagues [34]. This particular 2.5 h session was restructured in 2019/20 given a redesign in the overall undergraduate curriculum, when the vignette was not presented [23], and the COVID-19 pandemic started [35].

## Results and discussion

Our two outcomes, the vignette promoting an open dialogue around stigma and around mental health disorders, were believed to be achieved when considering the number of reflections submitted and the field notes taken representing in-class discussion. In fact, we received 148 reflections ranging from 202 to 301 words, and took de-identified field notes of students’ answers or responses to the vignette between 2015/16 and 2018/19, totaling 284 double-spaced pages of textual data. This manuscript focuses on the use of a vignette as an educational tool to promote open dialogue in general. It also focuses on the use of the vignette as a conducive mean to generate discussion and reflection within undergraduate DMD and BDSc-DH students who explored their own views on stigma around substance use and mental health disorders. The exploratory nature of the study lead to 6 major themes including: vignettes in undergraduate education; vignette to explore stigma and mental health disorders; the interplay of Link & Phelan’s stigma framework; patient-centered care approach to counteract stigma; vignette exploring power relations: and not a one-size-fits-all use of the vignette. Each of these themes are presented and discussed ahead.

### Vignettes in undergraduate education

The vignette was presented within an inverted and interactive classroom approach as contemporary teaching method as suggested by Vanka and colleagues [36]. In the UBC undergraduate dental curricula, vignettes and other forms of short narratives have been used to discuss issues of sexual diversity [37], community service learning [26], and ethics [38]. As per the vignette presented in Fig. 1 [3, 12], most students over the years felt it depicted an alternative way to facilitate the discussion of sensitive issues since health care workers, particularly oral health care providers, may not understand addiction and mental health disorders properly. Some students also commented on the potential use of the vignette to enable discussion and reflection of issues that emerged from lack of knowledge and awareness. Overall, students during this didactic session seemed to not lack knowledge or not to be unaware of issues around stigma. This could be because while such beliefs are thought to be held by the population in general [39], they tend to be less endorsed by those within higher levels of education. This premise, however, has not currently been the case with higher power – and presumed educated – political figures whose rhetoric only incites racism, stereotyping and discrimination [40–42].

### Vignette to explore stigma and mental health disorders

The vignette scenario aimed at exploring how students understood stigma and its components as potentially experienced by Mary (Fig. 1). Students have discussed stigma involving addiction and mental health disorders as the most often stigmatized conditions [43], but recognized that *“it sure does happen with other conditions … I guess almost anybody could be stigmatized at some point, for any reason outside the normally dictated or expected at the moment”* (24 year-old male dental student, 2015). In fact, issues of stigma based on certain characteristics or traits has been also experienced by other groups such as those who are overweight [44], those who are HIV positive [13, 15], and those who experience homelessness [17]. Other students, on the contrary, acknowledged the fact that stigma *“can manifest when the way people treat you or talk to you or behave around you is different than they would [treat] an everyday person … it can also be something that happens in a subtle manner, almost like a hidden prejudice”* (28 year-old female dental student, 2016). Variation in understanding stigma intertwined with prejudice and discrimination has been reported elsewhere [45], while their negative impact to those affected remains [12, 13, 15].

### The interplay of Link & Phelan’s stigma framework

The use of the vignette has helped students to discuss the fact that stigma can be very real and happen within detrimental emotions. In particular, Link & Phelan’s framework conferring labelling as an experience was mentioned by a 22 year-old female dental hygiene student from 2017 as *“the dentist in this scenario probably thought of this patient as being less than what she is … by assuming that her teeth had to be extracted and by labeling her as being an ‘addict’ regardless of her actual dental needs as a whole”*. In agreement with Link & Phelan, students’ views revealed that labels become the basis for stereotypes as they set into action negative images about certain conditions and individuals.

Some students’ comments reflected many misconceptions and stereotypes that society may hold against Mary and others alike, as also acknowledged by Link & Phelan’s stigma framework. In fact, Najman and coworkers [46] looked at stigmatizing characteristics of some patients in health care and demonstrated that both addiction and mental disorders rank amongst the top ten most stigmatized conditions. As found by Varas-Diaz and colleagues [14], those who stereotype individuals with substance use and mental health disorders see them as *blameworthy* and the sole culprit for their health problems, as voiced by the following student: *“would you not expect that progression on the oral disease leading to tooth loss in light of her behaviour and life choices?”* (24 year-old dental hygiene student in 2015). While it is common for oral health care professionals to criticize their patients for their poor oral hygiene and consequent dental diseases, many patients perceive such criticisms as a lack of empathy and understanding [47]. Blaming individuals for not having a healthy lifestyle is more problematic than beneficial. This stereotypical view that patients like Mary are unable or unwilling to change brings up labels such as lacking willpower, and culminates with power imbalance as described by Link & Phelan’s stigma framework [5].

### Patient-centered care approach to counteract stigma

To counteract stereotypes and labels about patients like Mary, it has been suggested that health professionals must be educated to consider patients’ history and environmental, societal and personal factors that led to addiction and mental health disorders before making any misjudgement [3, 17]. Such holistic understanding of a patient’s life condition falls within a patient-centred care approach as advocated by Bedos and colleagues [48] and others [49]. In fact, students voiced that perhaps they should listen closely to individuals who had undergone substance dependence so that they may view this issue differently, with more compassion:

*“I felt that this scenario made me think about addiction as I never thought before. Normally, when I think about addiction, I immediately think of the negative aspects like drug and alcohol abuse. People usually see ‘drug addicts’ differently or treat them differently. Now, I feel that I view addiction slightly differently. Some people do not choose to take drugs or choose to live a life as an addict; some of them just took a wrong turn and went downhill from there.”* (22 year-old female dental student, 2016).

Statements like the above one are powerful because they are in agreement with what drug users had expressed that addiction was not a choice but rather triggered by their life circumstances where abuse, violence and despair drove them to escape from the reality surrounding their lives as a way to numb their suffering [50, 51]. Such understanding of drug use fits well under a patient-centred care philosophy. In other words, the vignette may have helped to dispel the stereotype that substance users are *at fault* for their own situation:

*“While reading this [scenario], I have realized the importance of listening to the affected individuals so I do not judge them on the surface. I don’t think it is a matter of choice [to use drugs], but their life circumstances, and it is common for them to be neglected*” (25 year-old male dental student in 2018).

### Vignette exploring power relations

In terms of the patient-professional relationship, students recognized that *“there is always a power relationship that some dentists make sure is there, while others try to minimize it”* (21 year-old female, dental hygiene student in 2017). In fact, power is especially prominent in health care interactions as providers usually have the knowledge and skills, and most often, a perceived higher social status than many patients [52]. This imbalance may generate stress and anxiety in patients who may simply need to feel engaged into good communication, and perceive that rapport has been established between them and their providers when discussing decision making [53], and health care options [54, 55].

Other students, on the contrary, felt that the scenario represented an expected circumstance in which the patient was in a stage of life that the treatment proposed by the dentist was the only choice given prognosis and feasibility. Perhaps for students (and professionals) with this kind of perception, a patient-centred care approach is lacking. Even though the vignette was meant to elicit students’ views on stigma, it also helped them to nurture non-stigmatizing attitudes. For example, some students pointed out that although some health care providers including dentists might hold stigma, ‘*others may express care and understanding as their professional qualities to convey an environment of care and courtesy, kindness and willingness to help out those who struggle with these issues in life* (24 years-old male dental student in 2015)*.* In cases like this, continuing education courses may further reinforce the need for providers to be mindful and non-judgmental in a shared decision-making approach.

### Not a one-size-fits-all use of the vignette

Although some students seemed to have easily expressed their ideas and felt comfortable commenting on each other’s opinions, some might have felt the scenario intimidating and stayed quiet despite attempts to engage them equally [56]. Others might have felt that the vignette did not offer sufficient medical history and clinical information about the patient to suggest a proper treatment plan or to discuss the route taken by Mary’s previous dentist. In fact, one student did comment on the treatment decision of the dentist, in an attempt to justify what was done once certain issues about Mary were assumed:

*“If the patient doesn’t have a lot of motivation or she is still using substances and her oral hygiene is very poor then it could be justified that you may remove all teeth to prevent worsening.”* (28 year-old male, undergraduate dental student in 2017).

Health care professionals in general may use assumptions to wrongly justify treatment options as expressed by the above student. But some other students did raise the need for better listening to Mary so that a clearer understanding of the psychosocial problems that have led to her poor oral status can be achieved, as suggested by Freeman and Ismail [57] and others [58]. While information pertaining to Mary’s psychosocial environment could have been depicted in the vignette, some students were more focused on finding a clinical solution and almost disregarding Mary’s personal life situation:

*“I’ve seen dozens of patients in the same situation. If a patient would present to me on abused substances and was in pain you have to address the chief concern. You find out what’s bothering her and then you treat that. I don’t think you treat it with medications, I think you treat it with dental treatment and there are gonna be a few options for each tooth.”* (23 year-old female dental hygiene student in 2017).

Dentists, along with other health providers, are responsible for the difficult task of creating a balance between pain management and the abuse of pain medications, especially in light of the number of recent deaths by opioid overdose [59, 60]. In fact, the issue of patients abusing pain medication was brought up by a 25 year-old male undergraduate dental student in 2015:

*“It seems that she likes the dentist to prescribe her pain medication so maybe there is another kind of substance abuse that she has and I have to put that in my mind … she said he’s nice but doesn’t prescribe so it seems that she just wants that medication to be prescribed; she only had one goal.”*

From the above quote, it seems like some students still held on to strong beliefs about substance use and seeking behavior, and perhaps the vignette could have been written to prompt a more in-depth discussion on these beliefs.

The six major themes that surfaced from the written notes taken from the open in-class discussions and from the written reflections gathered over the 4-year period enabled us to address our research question attesting that an educational vignette, depicting a fictitious patient with a history of substance use and mental health disorders accessing dental care, can promote an open dialogue about stigma.

### Study limitations

Although the vignette appeared to be a conducive tool to generate discussion around issues of stigma related to substance use and mental health disorders, this study included a relatively small number of undergraduate DMD and BDSc-DH students from one university only. These students may not be a typical representation of future oral health care professionals, and generalizability of the findings is limited. The use of a non-traditional qualitative inquiry without a commonly described approach or framework and a coding analysis might have biased the conclusions, although we provided a discussion based on themes. The situation in which the vignette was presented may have been intimidating to some of the students and despite the efforts to encourage open dialogue, some may have been embarrassed or shy to share their opinions. It is also possible that certain feelings were not mentioned in order to avoid peer judgment. The questions we posed during the educational sessions were not validated despite being used for various years. Future studies should attempt to validate such questions to explore the extent to which they ask what they are supposed get at in a clear and unbiased manner. Another drawback was the cross-sectional aspect of the study without a follow up, and the fact that the reflections were not analysed according to the students’ demographics which might have over or under represented certain characteristics. Some students may have also associated substance used with mental health disorders as co-occurring despite the educational session making it clear that was not always the case. Furthermore, the vignette may have been perceived by the students as portraying an individual with other stigmatizing characteristics such as being poor, and receiving social assistance. In other words, the stigmatization that some students may have understood was not exclusively due to substance use and/or mental health disorders, further perpetuating a particular life circumstance. As results and discussion were combined, it might have made it challenging to determine what was found in this study versus what was reported in the existing literature, although we did promptly use the literature to refute or corroborate our findings. Further studies utilizing the vignette to promote dialogue about mental illness and substance use and how it relates to stigma with undergraduate dental and dental hygiene students from other universities are warranted.

## Conclusions

This study was designed to explore how the use of a vignette in an inverted classroom approach with undergraduate dental professional students could be conducive to generate discussions on issues of stigma related to substance use and mental health disorders. The research question “*how can an educational vignette, depicting a fictitious patient with a history of substance use and mental health disorders accessing dental care, promote an open dialogue about stigma?*” was answered positively as it seemed to have facilitated a generally high level of discussion and allowed some students to reflect on the different dimensions of providing care to a patient with a history of substance use and/or mental health disorders. It also enabled the identification of six major themes associated with the textual data. While this manuscript explored students’ views on stigma as potentially experienced by those with a history of substance use and mental health disorders while accessing oral health care, it is unknown the impact of such methodology when students are ready to serve their communities and may face patients like Mary. Future studies evaluating the use of the proposed vignette to the ethical and professional development of dental professionals are needed.

## Data Availability

Due to the sensitivity of the information shared via the reflections, only de-identified data can be made available upon request to the first author.

## Abbreviations

COVID: Coronavirus Disease
HIV/AIDS: Human Immunodeficiency Virus/Acquired Immunodeficiency Syndrome
MSc: Master of Science
PhD: Doctor of Philosophy
UBC: University of British Columbia

## Footnotes

1 In North America, those individuals interested in becoming a dentist enroll in an undergraduate dental program at a university. The majority of these individuals already possess a bachelor’s degree in disciplines that do not necessarily relate to health. The undergraduate dental degree is usually completed in four years, and the graduates will hold a Doctor of Dental Medicine (DMD) or a Doctor of Dental Surgery (DDS). For the dental hygiene students, they enroll in a bachelors’ degree in dental hygiene, following high school. Undergraduate dental hygiene education can take the form of a bachelor of dental science in dental hygiene (BDSc-DH), and some of these BDSc DH graduates can then apply to the DMD program.

2 The inverted classroom approach is a blended-learning method in which a self-directed learning component precedes the classroom instruction. The self-direct learning imparts the factual knowledge that serves as a basis for the classroom instruction used to further assimilate the previously gained knowledge. For the study reported herein, we employed self-directed pre-work, in-session activity with two didactic parts, small group discussion, and post-class reflection.
